# Anti-inflammatory and anti-oxidative effects of the green tea polyphenol epigallocatechin gallate in human corneal epithelial cells

**Published:** 2011-02-18

**Authors:** Megan E. Cavet, Karen L. Harrington, Thomas R. Vollmer, Keith W. Ward, Jin-Zhong Zhang

**Affiliations:** Global Pharmaceutical R&D, Bausch + Lomb, Rochester, NY

## Abstract

**Purpose:**

To determine the anti-inflammatory and anti-oxidant effects of epigallocatechin gallate (EGCG), the major polyphenol component of green tea, in human corneal epithelial cells (HCEpiC).

**Methods:**

HCEpiC were challenged with interleukin-1β (IL-1β) for 18 h or hyperosmolarity (440 mOsm) for 24 h. Luminex technology was used to determine the effects of EGCG (0.3 – 30 µM) on IL-1β- or hyperosmolar-induced cytokine release into the medium. Cell metabolic activity was measured using the alamarBlue assay. Effects of EGCG on mitogen-activated protein kinase (MAPK) phosphorylation were determined by cell-based enzyme-linked immunosorbent assay (ELISA) and western blotting. Effects of EGCG on nuclear factor kappa B (NFκB) and activator protein-1 (AP-1) transcriptional activity were assessed by reporter gene assay. The effects of EGCG on glucose oxidase (GO)-induced reactive oxygen species (ROS) production was determined using the ROS probe CM-H_2_DCFDA.

**Results:**

Treatment of HCEpiC with 1 ng/ml IL-1β for 18 h significantly increased release of the cytokines/chemokines granulocyte colony-stimulating factor (G-CSF), granulocyte-macrophage colony-stimulating factor (GM-CSF), interleukin-6 (IL-6), interleukin-8 (IL-8), and monocyte chemotactic protein-1 (MCP-1), while hyperosmolarity-induced release of IL-6 and MCP-1. When cells were treated with IL-1β and EGCG or hyperosmolarity and EGCG there was a dose-dependent reduction in release of these cytokines/chemokines, with significant inhibition observed at 3–30 µM. There was no effect of EGCG on cell metabolic activity at any of the doses tested (0.3–30 µM). EGCG significantly inhibited phosphorylation of the MAPKs p38 and c-Jun N-terminal kinase (JNK), and NFκB and AP-1 transcriptional activities. There was a significant dose-dependent decrease in GO-induced ROS levels after treatment of HCEpiC with EGCG.

**Conclusions:**

EGCG acts as an anti-inflammatory and anti-oxidant agent in HCEpiC and therefore may have therapeutic potential for ocular inflammatory conditions such as dry eye.

## Introduction

The corneal epithelium acts both as an initial physical barrier to insult and infection, and also as a key player in the ocular immune response by producing inflammatory cytokines [1]. Ocular surface inflammation can be induced by numerous insults including the presence of pathogens, allergic reaction, and dry eye disease. While acute inflammation is essential for healing, chronic inflammation contributes to epithelial damage and cell death [2].

Dry eye disease is associated with inflammation of the ocular surface, and hyperosmolarity of the tears is now recognized as an important mediator of this inflammatory response [3,4]. Increased tear osmolarity causes a signaling cascade which involves phosphorylation of the stress-activated mitogen activated protein kinases (MAPKs) p38 and c-Jun N-terminal kinase (JNK) [5-9], followed by activation of transcription factors such as activator protein-1 (AP-1) and nuclear factor kappa B (NFκB), resulting in increased levels of pro-inflammatory cytokines such as IL-1β, TNF-α, interleukin-8 (IL-8), and interleukin-6 (IL-6) [8-14]. Increased levels of IL-1β at the ocular surface contributes to the epithelial damage observed in dry eye and also infectious eye diseases with an inflammatory component by inducing expression of other pro-inflammatory cytokines in corneal epithelial cells and other ocular cells [15-17].

Another contributor to the cellular damage which occurs during inflammation is oxidative stress [18,19]. It has been demonstrated that lipid peroxide and myeloperoxidase activity are increased in the tears of dry eye patients, indicating increased oxidative potential [20]. In addition, increased oxidative stress markers and reactive oxygen species (ROS) production in the corneal epithelia of blink-suppressed dry eyes has been reported [21]. The ability of antioxidants to reduce the inflammatory reaction in acute inflammation and experimental infectious keratitis indicates that reactive oxygen species play a role in corneal inflammatory diseases [22,23].

Epigallocatechin gallate (EGCG) is the major polyphenol found in green tea, which is produced from the *Carmellia sinensis* plant. EGCG has been demonstrated to have both anti-inflammatory and anti-oxidant properties in multiple cell types [24-29]. Because of these properties, it is thought that EGCG may have therapeutic benefit in numerous inflammatory diseases such as atherosclerosis, arthritis, and dry eye disease [30-32]. Therefore, the objective of the current study was to determine the effects of EGCG on pro-inflammatory and pro-oxidant stimuli in human corneal epithelial cells (HCEpiC). Our data demonstrate that EGCG exhibits both anti-inflammatory and anti-oxidant properties in HCEpiC. To investigate the mechanistic basis for the anti-inflammatory effects of EGCG in HCEpiC, we determined its effect on the activation of MAPKs p38 and JNK and transcription factors NFκB and AP-1.

## Methods

### Reagents

EpiLife medium, human corneal growth supplement (HCGS), penicillin-streptomycin solution, alamarBlue solution, Lipofectamine LTX, and Opti-MEM were obtained from Invitrogen (Carlsbad, CA). EGCG was from DSM (Basel, Switzerland) and IL-1β was from R&D Systems (Minneapolis, MN). Human multiplex-cytokine kits were from Millipore (Billerica, MA). Fast Activated Cell-based ELISA (FACE) p38 and JNK ELISA kits were from Active Motif (Carlsbad, CA). Cignal AP1 reporter (luc) and Cignal NFκB reporter (luc) kits were purchased from SA Biosciences (Frederick, MD). Dual-Glo Luciferase assay system was from Promega (Madison, WI). All other reagents were purchased from standard commercial sources and were of the highest available purity.

### Cells and treatments

SV40-transformed human corneal epithelial cells (HCEpiC) were received at passage 20 from ATCC (Manassas, VA), and maintained in EpiLife medium supplemented with HCGS, 100 U/ml of penicillin, and 100 µg/ml of streptomycin at 37 °C in a humidified incubator with 5% CO_2_. Cells were cultured in glucocorticoid-free medium (EpiLife basal medium supplemented with 12.5 μg/ml bovine pituitary extract, 1.25 μg/ml bovine insulin, and 1.25 ng/ml EGF) for 48 h before exposure to IL-1β or hyperosmolar medium. Osmolarity was increased to 440 mOsM by the addition of 123 mM sucrose to basal EpiLife medium (at 317 mOsM). Osmolarity of the solutions was verified using an osmometer (Osmette; Advanced Intruments, Norwood, MA). EGCG stock solutions were prepared in DMSO. Each treatment was performed in at least triplicate, and appropriate dilutions were prepared to deliver a constant amount of the vehicle to each well. Lack of an effect of treatments on cell metabolic activity, an index of cell viability, was determined by the alamarBlue assay [33,34].

### Cytokine release by multiplex Luminex

HCEpiC were seeded on 48-well plates at a density of 2×10^4^ cells per well. After 72 h, cells were treated with vehicle (0.1% DMSO) or EGCG for 18 h with 1 ng/ml IL-1β or 24 h in 440 mOsM hyperosmolar basal media. Cytokine content in the culture medium was analyzed using multiplex Luminex bead technology [35,36] according to the manufacturer's instructions. Briefly, 25 μl of medium samples were incubated with antibody-coated capture beads overnight at 4 °C. Washed beads were further incubated with biotin-labeled anti-human cytokine antibodies for 1 h at room temperature followed by incubation with streptavidin-phycoerythrin for 30 min. Samples were analyzed using Luminex 200™ (Luminex, Austin, TX) and Statlia software (Brendan Technologies Inc., Carlsbad, CA). Standard curves of known concentrations of recombinant human cytokines were used to convert median fluorescence intensity (MFI) to cytokine concentration in pg/ml. Only the linear portions of the standard curves were used to quantify cytokine concentrations.

### Cell-based ELISA for determination of p38 and JNK phosphorylation

Cells were seeded on a 96-well plate at a density of 1×10^4^ cells per well. After 48 h, the medium was replaced with basal medium for 18 h. Due to the short incubation time necessary for measurement of MAPK phosphorylation, cells were pre-treated with EGCG for 2 h in basal medium. Cells were then treated with 1 ng/ml IL-1β alone or with EGCG at 3 or 30 μM, or hyperosmolar media alone or with EGCG at 3 or 30 μM for 30 min. Levels of phosphorylated and total p38 or JNK were measured using a cell-based ELISA according to the manufacturer’s instructions. Briefly, the cells were fixed with 4% formaldehyde in PBS. After blocking, cells were incubated with primary antibodies specific to the appropriate phosphorylated or total MAPK overnight at 4 °C. Negative controls were performed using secondary antibody only. After washing, cells were incubated with HRP-conjugated secondary antibody for 1 h followed by further washing. Chemiluminescent reagent was added to each well and the resulting luminescence was read on a Synergy plate reader (Biotek, Winooski, VT). To verify that cell number was unchanged by treatments, cells in each well were further stained with crystal violet. The absorbance was read on a Synergy plate reader at 595 nm. Data were expressed as the ratio of phosphorylated to total MAPK, and were normalized by cell number.

### Western blotting for determination of p38 and JNK phosphorylation

HCEpiC were preincubated with EGCG or vehicle control in EpiLife medium without growth factors for 2 h, and then were stimulated for 30 min with 1 ng/ml IL-1β. The cells were washed with ice-cold PBS and lysed in cell lysis buffer (62.5 mM Tris-HCl, pH 6.8, 2% SDS, 10% glycerol and 1:1,000 protein inhibitor cocktail [PIC; Sigma, St Louis, MO]). Cells were sonicated, centrifuged at 12,800× g, and protein amount was determined using a Micro BCA protein assay kit (Pierce, Rockford, IL). Aliquots of cell lysate (30 μg) were separated by SDS-polyacrylamide electrophoresis and transferred to nitrocellulose membranes for western analysis. Membranes were blocked with 5% BSA and exposed to phospho p38 or phospho JNK antibody (Cell Signaling, Danvers, MA). The blots were washed, and exposed to horseradish peroxidase (HRP) secondary antibody (Bio-Rad, Hercules, CA). After washing, blots were incubated in enhanced chemiluminescence signal (Pierce) and visualized using the Alpha Innotech Imaging system (Cell Biosciences, Santa Clara, CA). Blots were then stripped with Restore stripping solution (Pierce) and incubated in total p38 or JNK antibody, followed by HRP secondary antibody as a loading control.

### AP-1 and NFκB transcriptional activity

The AP-1 and NFκB transcriptional activities were measured using an inducible reporter construct which encoded a firefly luciferase reporter gene under the control of a basal promoter element (TATA box) joined to tandem repeats of specific AP-1 or NFκB transcriptional response elements. This vector was mixed with a constitutively expressing *Renilla* construct (which was used as a normalizer) at a ratio of 40:1. pRSVT-HCEpiC were seeded on a 96-well plate at a density of 1×10^4^ cells per well. After 24 h 200 ng of AP-1 or NFκB construct was transfected using Lipofectamine LTX transfection reagent (Invitrogen). Thirty h after transfection, cells were treated with vehicle (0.1% DMSO), IL-1β or 440 mOsm media, and 3 or 30 μM EGCG for 16 h in basal medium. Firefly and *Renilla* luciferase activity was measured using the Dual-Glo luciferase assay system (Promega). Background relative luminescence units (RLU) were subtracted and the ratio of firefly luciferase/*Renilla* luciferase luminescence was then calculated.

### Reactive oxygen species levels

The effect of EGCG on glucose oxidase-induced reactive oxygen species (ROS) generation was determined using the dye chloromethyl-2’,7’-dichlorodihydrofluorescein diacetate (CM-H_2_DCFDA). CM-H_2_DCFDA passively diffuses into cells, where it covalently binds to intracellular components, trapping the dye within the cell. Non-fluorescent CM-H_2_DCFDA becomes highly fluorescent when oxidized by ROS. Cells were pretreated with EGCG in basal EpiLife media for 1 h before dye loading. Cells were then loaded with 10 μM CM-H_2_DCFDA in basal EpiLife media for 30 min and washed with PBS. Cells were then exposed to vehicle, 50 mU glucose oxidase (GO), or GO + EGCG in EpiLife basal media for 1 h after which fluorescence was measured on a Synergy plate reader (BioTek, Winooski, VT). A control experiment in which EGCG was incubated with CM-DCF dye verified there was no effect of EGCG on the fluorescence emitted by the dye.

### Statistical analysis

Data were expressed as mean±SEM. Statistical analysis was performed using a one-way ANOVA-Dunnett’s test with the statistical software JMP8 (SAS Institute, Cary, NC). P values less than 0.05 were pre-determined to be statistically significant. Experiments were performed at least in triplicate.

## Results

### EGCG inhibits IL-1β- and hyperosmolar-induced cytokine release

**Figure 1:**
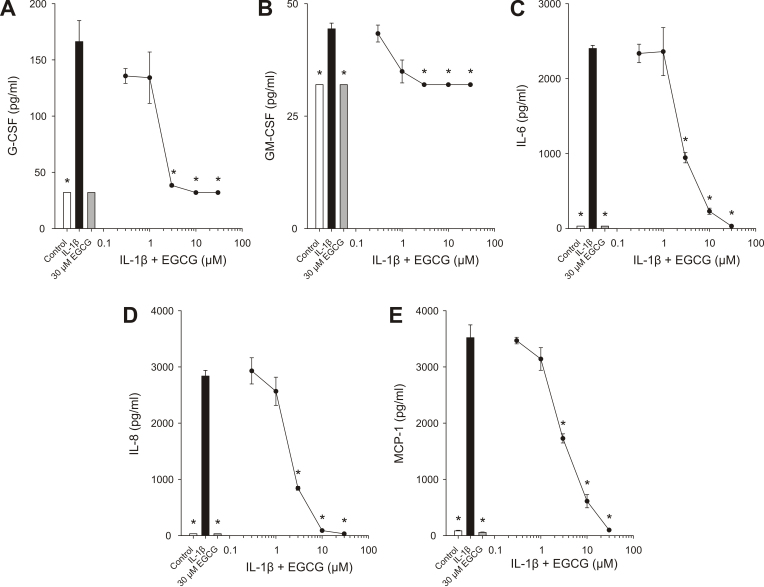
EGCG inhibits IL-1β-induced cytokine release in HCEpiC. Cells were cultured in complete (HCGS-containing) medium, followed by glucocorticoid-free medium for 18 h. Cells were then treated with 1 ng/ml IL-1β in the presence of EGCG for 18 h. G-CSF (**A**), GM-CSF (**B**), IL-6 (**C**), IL-8 (**D**), and MCP-1 (**E**) levels in the media were analyzed by Luminex. White bar represents control; black bar represents IL-1β; gray bar represents EGCG alone, black circles + line represent EGCG. Lines are the linear interpolation between data points. Data are presented as mean±SEM, n=3. *versus IL-1β; p<0.05.

Treatment of HCEpiC with 1 ng/ml IL-1β for 18 h significantly increased release of the cytokines/chemokines G-CSF, GM-CSF, IL-6, IL-8, and MCP-1. When cells were treated with IL-1β and EGCG there was a dose-dependent decrease in release of these cytokines/chemokines, with significant inhibition observed at 3 – 30 μM (Figure 1).

**Figure 2:**
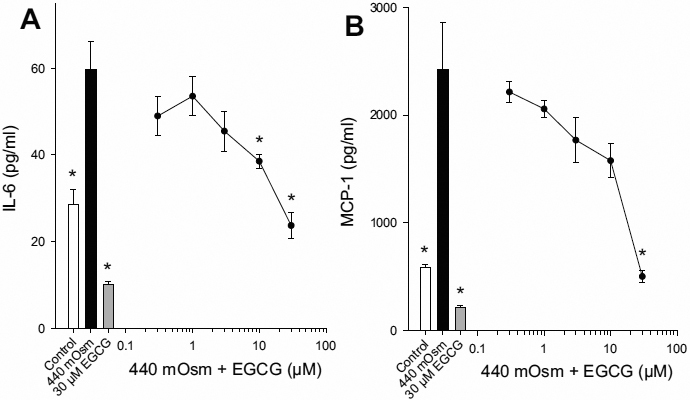
EGCG inhibits hyperosmolar-induced cytokine release in HCEpiC. Cells were cultured in complete (HCGS-containing) medium, followed by glucocorticoid-free medium for 18 h. Cells were then treated with 440 mOsm hyperosmotic basal media in the presence of EGCG for 24 h. IL-6 (**A**) and MCP-1 (**B**) release into the media was analyzed by Luminex. White bar represents control (317 mOsm); black bar represents hyperosmolarity (440 mOsm); gray bar represents EGCG alone; black circles + line represents EGCG. Lines are the linear interpolation between data points. Data are presented as mean±SEM, n=3. *versus 440 mOsm hyperosmotic media; p<0.05.

Increased tear osmolarity is a key factor in dry eye disease [3,4], therefore the effect of EGCG on hyperosmolar-induced cytokine release was determined. There was a significant increase in IL-6 and MCP-1 release when the medium osmolarity was increased from 317 mOsm to 440 mOsm for 24 h. There was a dose-dependent inhibition of hyperosmolar-induced IL-6 and MCP-1 release, with significant inhibition observed at 10 and 30 μM for IL-6 and 30 μM for MCP-1 (Figure 2A,B).

**Figure 3:**
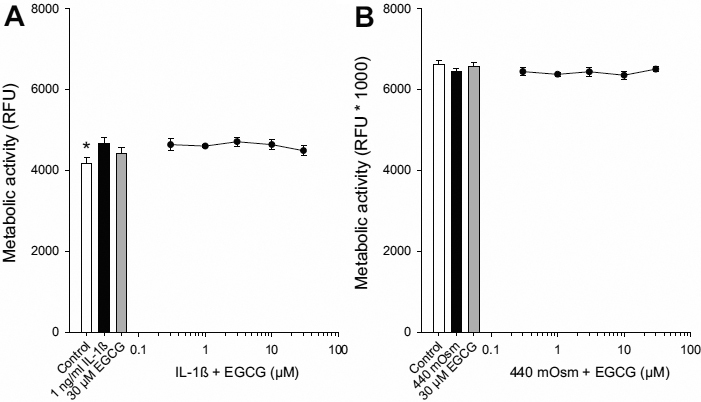
Effects of EGCG on cell metabolic activity in HCEpiC. Cells were cultured in complete (HCGS-containing) medium, followed by glucocorticoid-free medium for 18 h. Cells were then treated with IL-1β (**A**) or 440 mOsm hyperosmotic basal media (**B**) in the presence of EGCG for 18 or 24 h, respectively. Cell metabolic activity was determined using an alamarBlue assay. Data are presented as mean±SEM, n=3. *versus IL-1β for panel **A** and 440 mOsm media for panel **B**; p<0.05.

To verify that the inhibitory effect of EGCG on cytokine release was not due to cellular toxicity an alamarBlue assay was performed. There was a small, yet significant increase in metabolic activity in cells treated with IL-1β as compared to control (Figure 3A). However, there was no effect of IL-1β + EGCG or hyperosmolarity + EGCG on cell metabolic activity at any of the doses tested (0.3–30 μM; Figure 3A,B). These data demonstrate that EGCG can inhibit inflammatory cytokines induced by multiple stimuli in HCEpiC.

### EGCG reduces IL-1β- and hyperosmolar-induced phosphorylation/activation of p38 and JNK MAPK

**Figure 4:**
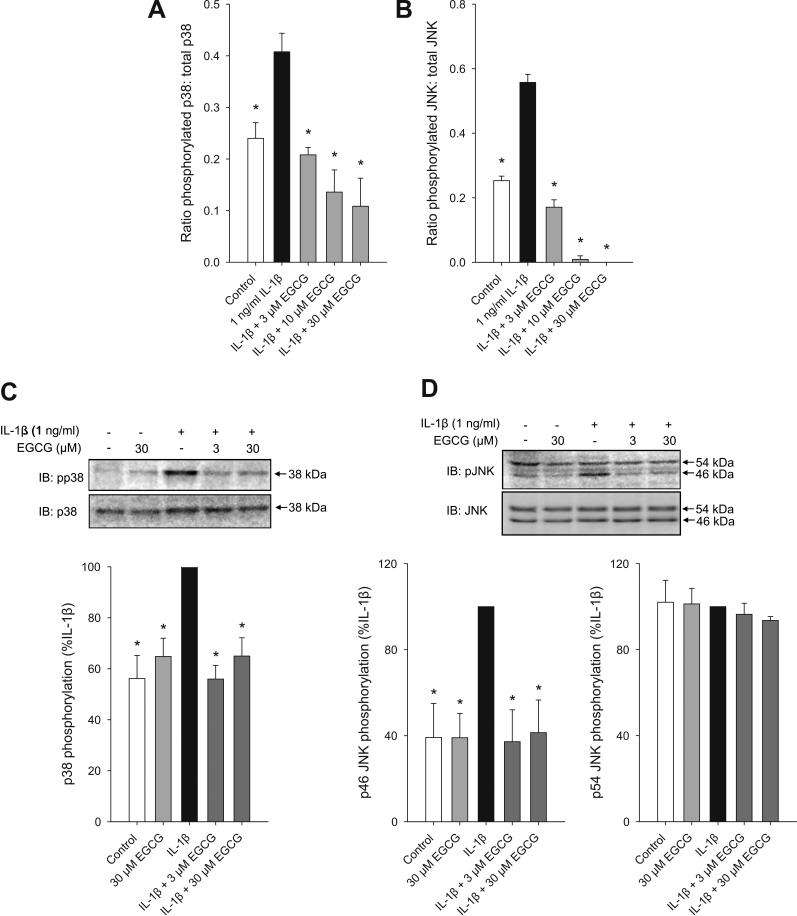
Effects of EGCG on IL-1β-induced phosphorylated p38 and JNK levels in HCEpiC. Cells were cultured in complete (HCGS-containing) medium, followed by basal medium for 18 h. Cells were pre-treated with EGCG for 2 h. Cells were then treated with IL-1β + EGCG for 30 min. Phosphorylated p38 and JNK levels were determined by cell-based ELISA (upper panels) or western blotting (lower panels). **A**: effect of EGCG on IL-1β-induced phosphorylated p38 determined by cell-based ELISA; **B**: effect of EGCG on IL-1β-induced phosphorylated JNK determined by cell-based ELISA; **C**: effect of EGCG on IL-1β-induced phosphorylated p38 determined by western blotting, Upper panel: upper blot shows phosphorylated p38. Lower blot is after stripping and probing with total p38 antibody. Lower panel: densitometric analysis of phosphorylated p38 normalized by total p38. D. effect of EGCG on IL-1β-induced phosphorylated JNK determined by western blotting, Upper panel: upper blot shows phosphorylated JNK. Lower blot is after stripping and probing with total JNK antibody. Lower left panel: densitometric analysis of phosphorylated p46 JNK normalized by total p46 JNK; lower right panel: densitometric analysis of phosphorylated p54 JNK normalized by total p54 JNK. For **A** and **B**, n=6, For **C** and **D**, n=3–4. Representative blots are shown. *versus IL-1β; p<0.05.

To investigate the possible mechanisms underlying the anti-inflammatory effects of EGCG, its effects on phosphorylation of the MAPKs p38 and JNK were determined. Quantitative cell-based ELISA and semi-quantitative western blotting were performed using phospho-specific antibodies against JNKs at Thr183/Tyr185, and against p38 at Thr180/Tyr182. These sites have been demonstrated to be associated with the activation state of these proteins [37]. Exposure of HCEpiC to 1 ng/ml IL-1β induced an increase in the ratio of phosphorylated to total p38 and JNK, indicating an increase in activity of these inflammatory signaling mediators (Figure 4). Figure 4A,B shows that both p38 and JNK phosphorylation, as measured by cell-based ELISA and normalized to total p38 or JNK, were significantly inhibited by 3, 10, or 30 μM EGCG. Similar results were obtained using western blotting techniques. Both 3 and 30 μM EGCG significantly inhibited phosphorylation of IL-1β-induced p38 and p46 JNK with no change in total p38 or p46 JNK expression, however p54 JNK phosphorylation was unaltered by 1 ng/ml IL-1β or EGCG (Figure 4C,D).

**Figure 5:**
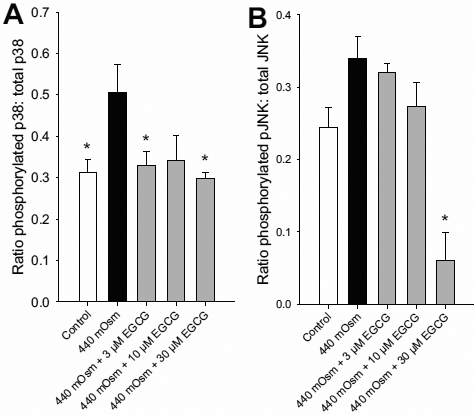
Effects of EGCG on hyperosmolar-induced phosphorylated p38 and JNK levels in HCEpiC. Cells were cultured in complete (HCGS-containing) medium, followed by basal medium for 18 h. Cells were pre-treated with EGCG for 2 h. Cells were then treated with 440 mOsm hyperosmotic media + EGCG for 30 min. Phosphorylated p38 and JNK levels were determined by cell-based ELISA. **A**: effect of EGCG on hyperosmolar-induced phosphorylated p38; **B**: effect of EGCG on hyperosmolar-induced phosphorylated JNK; n=6, *versus 440 mOsm media; p<0.05.

The effect of EGCG on hyperosmolar-induced p38 and JNK phosphorylation was also determined by cell-based ELISA. There was a significant increase in p38 phosphorylation when cells were exposed to hyperosmolarity for 30 min, however the JNK phosphorylation increase did not reach significance. Hyperosmolar-induced phosphorylation of p38 was significantly decreased when cells were treated with 3 and 30 µM EGCG, as compared to hyperosmolarity alone (Figure 5A). There was also a significant inhibition of JNK phosphorylation at 30 μM EGCG (Figure 5B).

### EGCG suppresses IL-1β- and hyperosmolar-induced AP-1 and NFκB transcriptional activation

**Figure 6:**
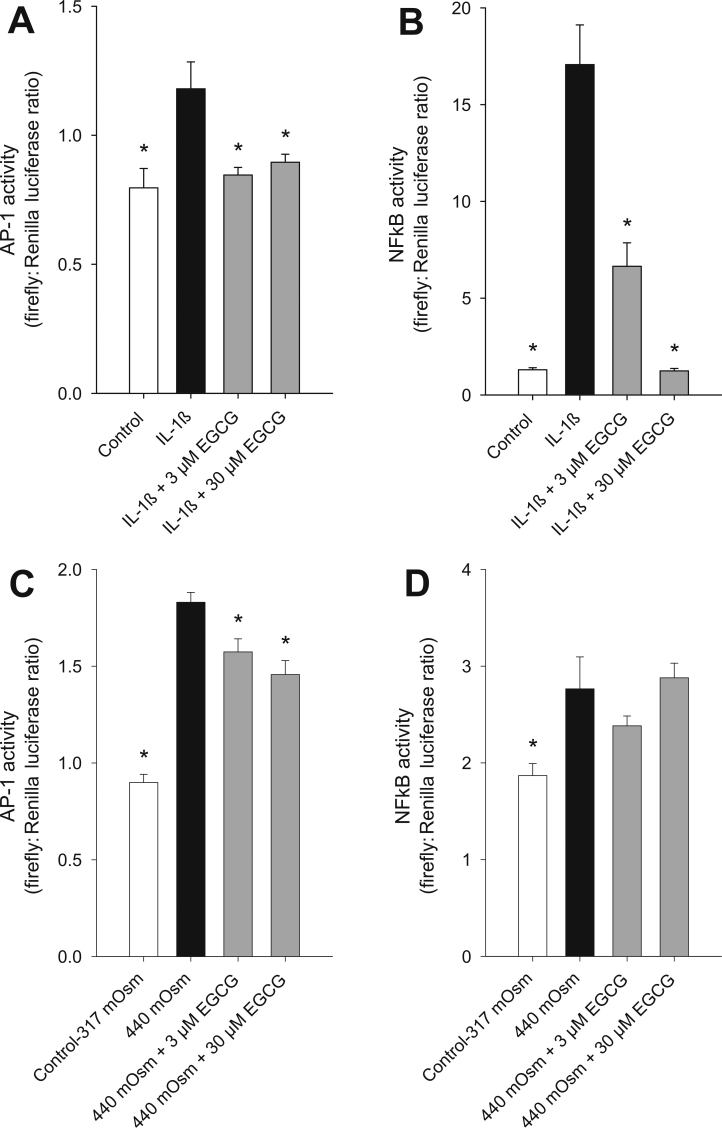
Effects of EGCG on IL-1β- and hyperosmolarity-induced HCEpiC NFκB and AP-1 activity. Cells were transfected with an AP-1 or NFκB reporter gene (expressing firefly) and a constitutively expressing *Renilla* construct and then placed in glucocorticoid-free medium for 24 h. Thirty h after transfection, cells were treated with IL-β (panels **A** and **B**) or 440 mOsM media (panels **C** and **D**) in the presence or absence of EGCG. Cells were assayed for firefly luciferase followed by *Renilla* luciferase luminescence 48 h after transfection. Data are expressed as normalized firefly: *Renilla* luciferase ratio. **A**: Effect of EGCG on IL-1β-induced AP-1 transcriptional activity; **B**: effect of EGCG on IL-1β-induced NFκB transcriptional activity. **C**: Effect of EGCG on 440 mOsm hyperosmolar-induced AP-1 transcriptional activity; **B**: effect of EGCG on 440 mOsm hyperosmolar-induced NFκB transcriptional activity. Data are mean±SEM, n=6. For **A** and **B**, *versus IL-1β; for **C** and **D**, *versus 440 mOsm media; p<0.05.

It has been reported that MAPK phosphorylation leads to activation of the transcription factors AP-1 and NFκB, and that furthermore, EGCG treatment leads to AP-1 and NFκB suppression in many cell types [30,31,38]. Therefore, the effect of EGCG on activation of AP-1 and NFκB was determined in HCEpiC. Luciferase reporter gene constructs containing tandem repeats of NFκB or AP-1 specific transcriptional response elements were transiently transfected into HCEpiC together with a constitutively expressing *Renilla* construct as a normalizer. Cells were then treated with 1 ng/ml IL-1β (Figure 6A,B) or 440 mOsm hyperosmolarity (Figure 6C,D) in combination with EGCG. Figure 6A,B shows that NFκB and AP-1 transcriptional activities were significantly increased by IL-1β. EGCG at 3 or 30 μM reduced this IL-1β-induced transcriptional activity to control levels. Similarly, hyperosmolarity induced both AP-1 and NFκB activities (Figure 6C,D). Hyperosmolar-induced AP-1 activity was significantly decreased by 3 and 30 μM EGCG, while there was no inhibition of NFκB activity.

### EGCG inhibits glucose oxidase-induced ROS in HCEpiC

**Figure 7:**
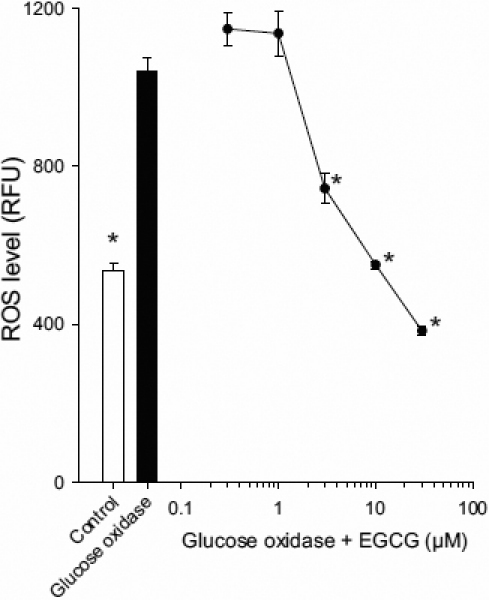
Effect of EGCG on glucose oxidase-induced ROS levels in HCEpiC. Cells were cultured in complete (HCGS-containing) medium, followed by glucocorticoid-free medium for 18 h. Cells were pretreated with EGCG for 1 h, loaded with CM-H_2_DCFDA for 30 min, followed by treatment with 50 mU/ml glucose oxidase and EGCG for 1 h. White bar represents control; black bar represents glucose oxidase (GO) alone; black circles indicate glucose oxidase and EGCG. n=4, *versus GO, p<0.05.

EGCG is a well known anti-oxidant in many cell types, and since oxidative stress is a key component of the inflammatory cascade, the effect of EGCG on ROS in HCEpiC was determined. The ROS generator glucose oxidase significantly increased ROS levels in HCEpiC after 1 h, as determined using CM-H_2_DCF-DA dye. There was a dose-dependent inhibition of glucose oxidase-induced ROS, with significant inhibition observed at 3, 10, and 30 μM EGCG (Figure 7), demonstrating that EGCG acts as an anti-oxidant in HCEpiC.

## Discussion

In this study we determined that the green tea polyphenol EGCG has both anti-inflammatory and anti-oxidant properties in HCEpiC. Two pro-inflammatory stimuli were selected to challenge the HCEpiC, IL-1β, and hyperosmolarity. The importance of IL-1β in the etiology of corneal inflammatory disease such as dry eye and keratitis has been demonstrated in multiple cell types [15-17,39-41]. An increase in osmolarity of the tear film is known to be one of the causative factors in dry eye, resulting in inflammation and subsequent cell damage [3,4]. In addition, studies have demonstrated that increased osmolarity elevates levels of cytokines and matrix metalloproteases in corneal epithelial cells [5-7], in in vivo dry eye models [8,9] and in the tears of dry eye patients [11-14]. EGCG was effective in inhibiting multiple cytokines induced by both these inflammatory mediators, demonstrating it may have therapeutic potential in the treatment of corneal inflammatory diseases, including dry eye. These data are in agreement with an in vivo study which demonstrated that topical EGCG was efficacious for the treatment of dry eye disease [32]. Furthermore, we demonstrate that EGCG exhibited potent anti-oxidant activity in HCEpiC, inhibiting reactive oxygen species generated by the pro-oxidant glucose oxidase.

EGCG is reported to possess both anti-inflammatory and anti-oxidant properties in vitro and in vivo [24-27,30-32]. In the eye, green tea extract protects the lens and retina from UV-induced oxidation [28,29,42] and reduces retinal oxidative glutamate toxicity [43]. While the anti-inflammatory and anti-oxidant properties of EGCG are well described in multiple ocular and non-ocular cell types, to our knowledge, this is the first study demonstrating these properties and their mechanistic basis in corneal epithelial cells. The stress-activated MAPKs p38 and JNK are known to play a key role in regulating the production of pro-inflammatory cytokines leading to inflammation. EGCG has been shown to inhibit both JNK and p38 in multiple cell types [31,44]. We found that EGCG inhibited the IL-1β- and hyperosmolar-induced phosphorylation/activation of both p38 and JNK in HCEpiC. EGCG was comparably potent at inhibiting both IL-1β-induced p38 and JNK activation, with significant inhibition at 3 μM. However, EGCG appeared more efficacious at inhibiting hyperosmolar-induced p38 phosphorylation than JNK, with significant inhibition at 3 μM for p38 as compared to 30 μM for JNK.

Two transcription factors known to mediate the increases in cytokine production by both IL-1β and hyperosmolarity are AP-1 and NFκB [45,46]. AP-1 is regulated by multiple MAPKs – ERKs activate AP-1 after mitogenic stimulation of cells, in contrast JNK and p38 activate AP-1 after exposure of cells to environmental stress or proinflammatory cytokines [47]. NFκB is commonly made up of a heterodimer between the p65 subunit (also known as Rel A), which contains a transactivation domain mediating transcriptional induction and the p50 subunit. NFκB is predominantly activated through phosphorylation by inhibitory protein kappa B (IκB) kinase and subsequent degradation of the bound IκB, allowing it to translocate to the nucleus [2]. Our results indicate that inhibition of IL-1β-induced cytokine expression by EGCG is mediated, at least in part, by inhibition of both AP-1 and NFκB transcriptional activities. Inhibition of AP-1 activity by EGCG is presumably due to its effect on MAPK signaling and EGCG may also affect DNA binding activity of AP-1 as has been demonstrated in other cell types [31,48]. The effects of EGCG on NFκB in other cell types are through multiple mechanisms, including inhibition of IκB kinase, IκB phosphorylation, p65NFκB phosphorylation, p65NFκB acetylation, and NFκB DNA binding activity [31,48,49].

While hyperosmolar-induced AP-1 activity was inhibited by hyperosmolarity, NFκB activity was not affected by EGCG. Incomplete inhibition of AP-1 activity and lack of effect on NFκB activity by EGCG suggests that EGCG inhibits hyperosmolar-induced cytokine levels at additional steps in the hyperosmolarity-induced signaling cascade. For example, the transcription factor nuclear factor of activated T-cell-5 (NFAT5) is induced and activated in limbal epithelial cells by hyperosmolarity [50]. Signal transducer and activator of transcription (STAT1) is another factor known to be activated by hyperosmolarity in several cell types [51,52], is expressed in human corneal epithelial cells [53], and is also a target of EGCG [54-56]. Another possibility is that EGCG could down-regulate cytokine gene expression at the post-transcriptional level through effects on mRNA stability [57]. Elucidating the additional mechanisms by which EGCG affects hyperomolar-induced cytokine release is an interesting area for future research.

In summary, these data demonstrate EGCG acts as an anti-inflammatory and anti-oxidant agent in HCEpiC challenged with either the pro-inflammatory cytokine IL-1β or hyperosmolarity. Therefore, EGCG has potential therapeutic application for the treatment of ocular disorders with an inflammatory component, including dry eye.
